# Computed Tomography-Based L1 Bone Mineral Density in 624 Dutch Trauma Patients—Are North American Reference Values Valid in Europe?

**DOI:** 10.3390/jpm12030472

**Published:** 2022-03-16

**Authors:** Tim Kobes, Arthur Sweet, Sophie Verstegen, Marijn Houwert, Wouter Veldhuis, Luke Leenen, Pim de Jong, Mark van Baal

**Affiliations:** 1 Department of Surgery, University Medical Center Utrecht, P.O. Box 85500, 3508 GA Utrecht, The Netherlands; a.a.r.sweet-3@umcutrecht.nl (A.S.); s.b.h.verstegen@students.uu.nl (S.V.); r.m.houwert@umcutrecht.nl (M.H.); l.p.h.leenen@umcutrecht.nl (L.L.); m.c.p.vanbaal-5@umcutrecht.nl (M.v.B.); 2 Department of Radiology, University Medical Center Utrecht, P.O. Box 85500, 3508 GA Utrecht, The Netherlands; w.veldhuis@umcutrecht.nl

**Keywords:** bone mineral density, computed tomography, reference values, first lumbar vertebra

## Abstract

Opportunistic screening for bone mineral density (BMD) of the first lumbar vertebra (L1) using computed tomography (CT) is increasingly used to identify patients at risk for osteoporosis. An extensive study in the United States has reported sex-specific normative values of CT-based BMD across all ages. The current study aims to validate North American reference values of CT-based bone mineral density in a Dutch population of level-1 trauma patients. All trauma patients aged 16 or older, admitted to our level-1 trauma center during 2017, who underwent a CT scan of the chest or abdomen at 120 kVp within 7 days of hospital admission, were retrospectively included. BMD measurements in Hounsfield Units (HU) were performed manually in L1 or an adjacent vertebra. Student’s *t*-tests were performed to compare the Dutch mean BMD value per age group to the North American reference values. Linear regression analysis and Pearson’s correlation coefficient (ρ) calculations were performed to assess the correlation between BMD and age. In total, 624 patients were included (68.4% men, aged 16–95). Mean BMD decreased linearly with 2.4 HU per year of age (ρ = −0.77). Sex-specific analysis showed that BMD of premenopausal women was higher than BMD of men at these ages. Dutch mean BMD values in the age groups over 35 years were significantly lower than the North American reference values. Our findings indicate that using North American BMD thresholds in Dutch clinical practice would result in overdiagnosis of osteoporosis and osteopenia. Dutch guidelines may benefit from population-specific thresholds.

## 1. Introduction

In Western countries, osteoporosis leads to an increasing clinical and economic burden driven by the aging population [1,2]. A recent study in European countries showed that osteoporosis management has not improved sufficiently in the past years, as fracture-related costs and the treatment gap has increased [3]. In the absence of a true gold standard, osteoporosis is most often diagnosed by assessing bone mineral density (BMD) of the femoral neck or lumbar spine using dual-energy X-ray absorptiometry (DXA) [4,5]. Many patients at risk for osteoporosis are overlooked, as screening is recommended mainly in the elderly or in patients who already sustained an adult age fracture [6].

In the past decade, there has been a trend towards opportunistic screening of BMD using CT images that were acquired for other clinical purposes by measuring the mean Hounsfield Unit (HU) density of trabecular bone in the first lumbar vertebra (L1) [7,8]. These HU measurements correlate significantly with T-scores as calculated with DXA measurements, and therefore provide an alternative method for assessing BMD with no additional radiation exposure or costs [8,9]. Moreover, CT imaging is increasingly considered a more accurate tool for BMD assessment in comparison to DXA, as was shown, for example, in trauma and oncologic patients [10,11]. Furthermore, BMD assessment using DXA has been shown to suffer from misguided elevation caused by vertebral compression fractures, degenerative joint disease, and vascular calcifications, which could be avoided using CT [12].

Different thresholds for defining osteoporosis based on studies on the correlation between L1 trabecular attenuation and DXA T-scores have been reported, with the largest study recommending a threshold of 135 HU [7,12]. In addition to these cut-off values, an extensive study on CT-based L1 attenuation in over 20,000 patients in the United States suggested sex-specific normative values across all ages using routine CT images made for a variety of indications [13]. As these values were aimed to serve as a reference for identifying patients at risk for osteoporosis, which may consequently lead to treatment, validation is required to determine whether these North American normative ranges generalize to other populations [13,14].

Therefore, the current study aims to validate North American reference values of computed tomography-based bone mineral density in a Dutch population of level-1 trauma patients.

## 2. Materials and Methods

### 2.1. Study Population

The medical ethical committee at our institution approved this cross-sectional study, and informed consent was waived. The study cohort consisted of all consecutive trauma patients aged 16 or older admitted to the University Medical Center Utrecht (UMCU) in the Netherlands, a level-1 trauma center, during 2017. Patients that underwent a CT scan of the abdomen, lumbar spine, or aorta, within 7 days of hospital admission were retrospectively included. Patients were excluded if neither L1, L2, nor the 12th thoracic vertebra (Th12) was intact and assessable or if CT imaging was not performed at 120kVp. In case more than one CT scan was performed during admission, the first was used for BMD assessment. Age, sex, American Society of Anesthesiologists (ASA) classification, mechanism of injury (MOI), and anonymized CT images were obtained for all patients.

### 2.2. Radiologic Assessment

Analysis was performed on CTs acquired on either a 64 or 256 detector row scanner (Philips Medical Systems, Cleveland, OH, USA). Administration of intravenous contrast was used in case of an abdominal scan in a trauma patient or angiographic CT scans of the aorta.

Measurements of BMD were performed manually by three investigators (TK, AS, SV) using PACS IDS7 21.1.2 (SECTRA) as described by Jang et al. [13]. In brief, a circular region of interest (ROI) was placed as centered as possible in the trabecular bone between the upper surface of the vertebra and the basivertebral vein. In the case of intravenous contrast administration, the split bolus technique with an interval of approximately 70 seconds was used to assess both the portal and the arterial phase simultaneously. Borderline cases were discussed with a senior staff radiologist (PdJ). In sixty randomly selected patients, BMD assessment was performed by all three investigators and the senior staff radiologist to assess interobserver agreement. The resulting intraclass correlation coefficient was 0.96. In patients with no assessable L1 measurement, BMD was measured at the level of Th12 or L2 instead, respectively. Preferably, an axial slice thickness of 0.9-mm was used; otherwise, axial slices of up to five-millimeter thickness were utilized.

Pickhardt et al. showed that BMD at the thoracolumbar junction and in the lumbar vertebrae decreases caudally. Consequently, the recommended osteoporosis threshold declines with five HU per vertebra in Th12 to L2 [7]. In our cohort, a correction was made when Th12 or L2 was measured instead of L1, based on the findings of Pickhardt et al. To create an equivalent BMD_L1_ value, five HU were pragmatically subtracted from BMD_Th12_, and five HU were added to BMD_L2_.

### 2.3. Statistical Analysis

Statistical analysis was performed using R 1.3.1093 for Mac (© the R Foundation for Statistical Computing, 2019). Mean BMD values and standard deviations (SDs) per age group from Jang et al. were used as the North American reference cohort [13]. Patients in the Dutch cohort were divided into age groups (<30, >85, and each 5-year interval in between). BMD was assumed to be normally distributed and described using the mean and SD. Not normally distributed variables were described using the median and interquartile range, dichotomous variables in proportions. Differences between BMD in the Dutch cohort and the North American reference cohort were compared per age group using the Student’s *t*-test. As Jang et al. did not measure all BMDs manually and described significant differences between automatically and manually measured BMD values, the BMD values of the Dutch reference cohort were compared with the manually measured North American reference values. Sex-specific differences in BMD between the Dutch and North American cohorts were assessed using the Student’s *t*-test as well. Unfortunately, Jang et al. did not report the manual BMD values per sex and the overall mean BMD and SD were used for analysis. Linear regression analyses were performed to assess the decrease in BMD that is associated with age in the total population and per sex. Subsequently, concurrent correlations between BMD and age were assessed using Pearson’s correlation coefficient (ρ). A *p*-value < 0.05 was considered statistically significant.

## 3. Results

**Figure 1:**
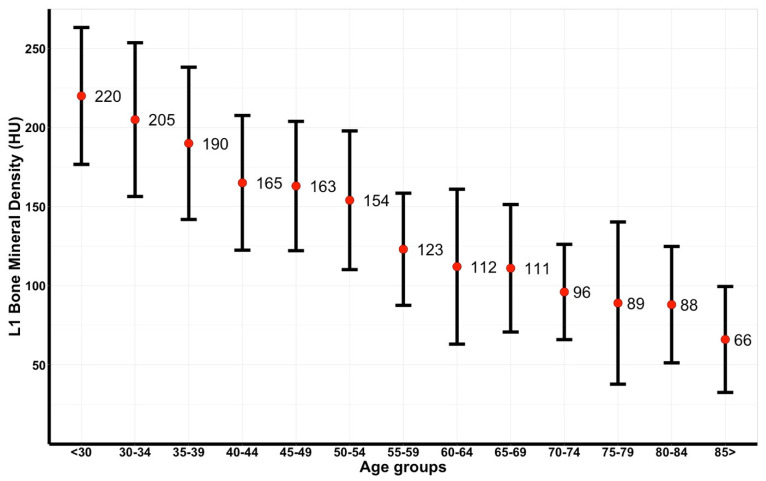
Errorbar plot of bone mineral density (in Hounsfield Units [HU]) in the total Dutch cohort, displayed in mean and standard deviation.

A total of 666 trauma patients underwent a relevant CT scan within seven days before or after UMCU admission during 2017. After excluding 30 patients due to exposure settings different than 120 kVp, and 12 patients due to non-assessable vertebrae, 624 patients were included. Almost one-third (31.6%) were women. Most BMD values could be derived from L1 (92.6%), with the remaining values being measured at the level of Th12 (5.6%) and L2 (1.8%). In 92.0% of cases, intravenous contrast agent was administered. Patients were mainly admitted after a motorized vehicle crash (32.2%), bicycle accident (20.0%), or low-energetic fall (20.8%). The mean BMD in the entire cohort was 152 ± 66 HU (Table 1). mAs values ranged from 23 to 294 (median 99). Patients were evenly distributed over the age groups, with the smallest group size of 26 patients (Table 2). The age group younger than 30 years consisted of 141 patients. The mean BMD per age group decreased gradually with age, with 220 ± 43 HU in the age group under 30 years and 66 ± 33 HU in patients above 85 years of age (Figure 1).

**Figure 2:**
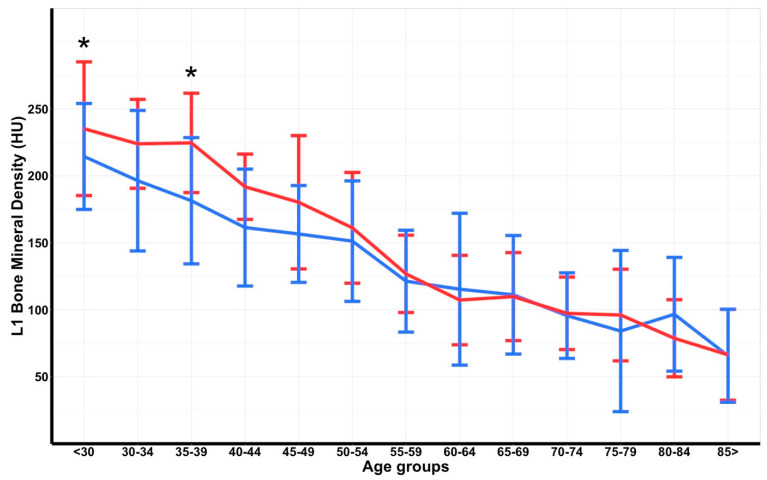
Errorbar plot of bone mineral density (in Hounsfield Units [HU]), displayed in mean and standard deviation, with comparison line graphs of means per 5-year interval showing men (blue) versus women (red) in the Dutch trauma cohort. The asterisk indicates statistical significance.

The BMD in women in the age groups <30 and 35–39 showed a significantly higher mean BMD compared to men at these ages (Table 2). In the sex-specific line graph of BMD decrease by age, women aged 30–34 and 40–49 had an overall non-significant higher BMD than men in these age groups, with the mean BMDs coinciding at the age group of 50–54 years (Figure 2).

**Figure A1:**
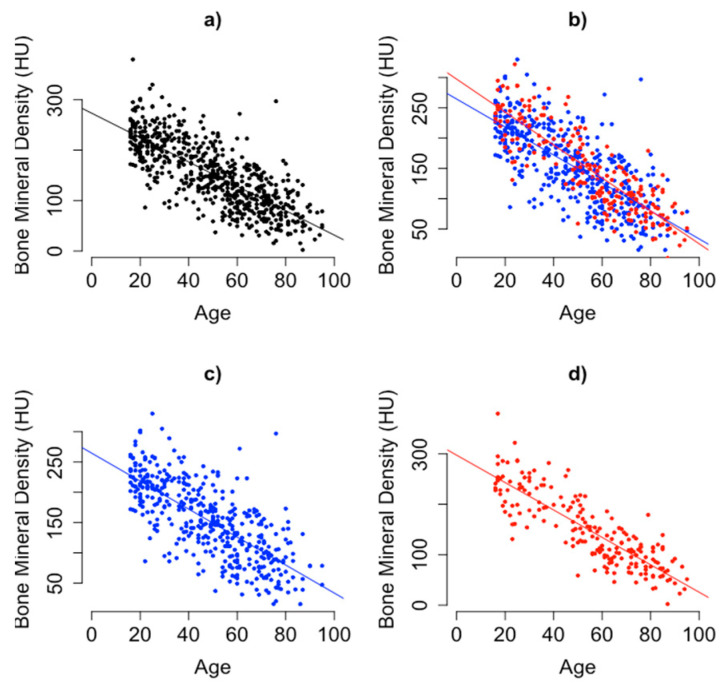
Scatterplots with linear regression lines of bone mineral density in Hounsfield Units (HU) compared to age in (**a**) the total cohort, (**b**) the total cohort differentiated by gender (men in blue, women in red), (**c**) only men, and (**d**) only women.

BMD was strongly correlated with age (ρ = −0.77): more in women (ρ = −0.85) than in men (ρ = −0.73) (Table A1; Figure A1). Linear regression analysis showed a significant decrease of BMD in the total population of 2.4 HU per year of age (Table A1; Figure A1). The significant sex-specific decreases of BMD were 2.3 per year in men and 2.7 per year in women when assessed over all age groups (Table A1; Figure A1).

**Figure 3:**
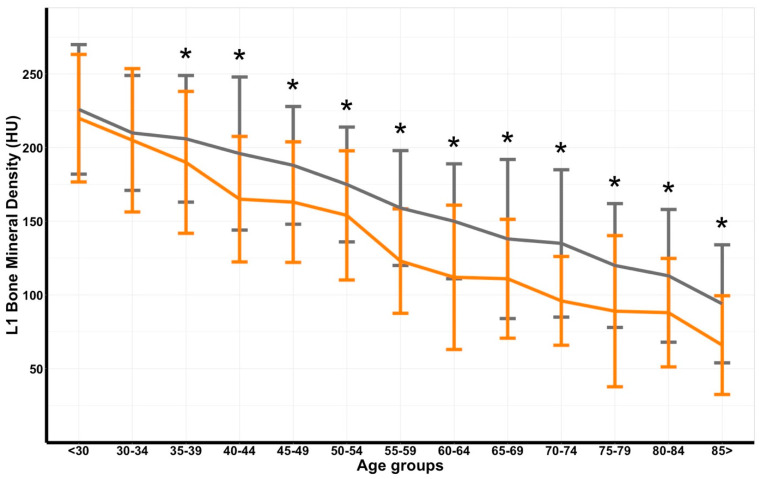
Errorbar plot of bone mineral density (in Hounsfield Units [HU]), displayed in mean and standard deviation, with comparison line graphs of means per 5-year interval showing the Dutch trauma cohort (orange) versus the North American reference cohort (gray) [13]. The asterisk indicates statistical significance.

Compared to the North American reference cohort by Jang et al., the Dutch cohort demonstrated a significantly lower mean BMD in all age groups, except in patients aged under 35 (*p*_<30_ = 0.189; *p*_30–34_ = 0.505) (Table 3; Figure 3) [13]. In men, the BMD difference between the Dutch and North American cohorts was significantly different in all age groups starting at the 40–44, except for the age group over 85. In women, the cohorts significantly differed as well starting at the post-menopausal age, except for the age group of 75–79 (Table A2).

## 4. Discussion

The current study presents L1 bone mineral density values on 120 kV computed tomography images in Dutch trauma patients. To our knowledge, this is the first study to provide these L1 values from a European population and compare those values to the North American reference data [13]. Our findings suggest that Dutch/European patients will benefit from dedicated, population-specific reference values and that care should be taken when using extrapolated North American values as thresholds for diagnosis.

Bone mineral density strongly correlated with age in this Dutch trauma cohort and showed a gradual decrease over the age groups. Linear regression analysis in the total population showed a decrease in BMD of 2.4HU per year of age. Sex-specific analysis showed that women aged <30 and 35–39 had a significantly higher mean BMD as compared to men in those age groups. At the age of 50 years and above, the mean BMD values of men and women were comparable. The HU decline in (post)menopausal women was to be expected as trabecular bone deposition decreases due to hormonal changes [15]. The values established in our study can be used as a starting point for future research on dedicated, BMD-determined reference values to diagnose osteoporosis in Dutch patients, who are at risk for adverse events such as fractures and increased mortality [16,17,18,19].

Bone mineral density values in the cohort of Dutch trauma patients were significantly lower in all age groups above 35 years as compared to the North American reference values [13]. A similar gradual decrease over the age groups was seen in both cohorts, yet with lower BMD values in the trauma cohort. Unfortunately, the mean values stated in this study are not yet suited for guidelines due to the small sample size. Moreover, an almost identical trend in sex-specific analysis, in which mean BMD in men and women differed at a younger age and coincided at the menopausal age, was reported by Jang et al. [13]. Due to the small sample size, mainly among the females, the power of our cohort most likely is not sufficient to reach statistical significance; however, no power analysis was performed. Nonetheless, the post-menopausal age groups were significantly different between both cohorts. This difference, however, could be due to the use of automatically measured BMD values in the North American cohort that were significantly higher in the study by Jang et al. [13]. Yet, the same decrease in BMD of 2.4HU per year of age found in the current study was reported in the North American cohort. In conclusion, Dutch trauma patients demonstrated a similar decreasing trend in BMD over age in the total population and per sex, yet with values significantly lower than the North American reference cohort.

When comparing the current results to the reference values of the North American cohort, several factors should be taken into account. First, in terms of the study population, there may exist baseline differences between the patients included in the Dutch cohort versus the North American cohort that were not reported in the present study, nor by Jang et al. [13]. The Dutch cohort consisted of level-1 trauma patients, whereas the North American reference cohort included patients who underwent a CT for a multitude of conditions and indications [13]. As bone mineral density is considered a frailty marker, one would expect a lower BMD in the Jang et al. cohort [20]. Therefore, we hypothesize that trauma patients are a proper representation of the normal Dutch population, and our results indicate an actual difference between both cohorts. However, with 31.6% of the study population being female, women were underrepresented in the current study. Second, there might be ethnic or racial differences between the populations that were compared in this study. A study comparing DXA-based BMD of white, African American, and Mexican American adults showed significant differences between all three populations [21]. We described Dutch patients predominantly; however, we did not register any other background information except age and sex. Jang et al. reported that their population was primarily white (90%), and we, therefore, believe that our cohorts are comparable in terms of race [13]. Third, there might be differences in weight or body mass index (BMI) of the included patients in the present study compared to the American cohort. Studies have shown that obesity affects BMD as measured with DXA, yet it is not entirely clear how obesity affects CT-based BMD [21,22,23,24]. This effect may be negligible, as three small studies did not demonstrate any significant association between weight or BMI and CT-based L1 trabecular attenuation [24,25,26]. Additional research is needed to assess the effect of obesity on BMD further. Fourth, we measured trabecular attenuation of L1 in most patients, but in case L1 was not assessable trabecular attenuation at Th12 or L2 was measured. As it was shown that BMD in these three vertebrae caudally decreases, a corrected BMD_L1_ was created [27]. Jang et al. acknowledged that using Th12 or L2 as a substitute for L1 is acceptable; however, these examinations were excluded from their reference cohort [13]. Fifth, in most of the Dutch trauma patients, an intravenous contrast agent was administered, which has been shown to increase the HU measured in L1 trabecular attenuation [28,29,30,31]. Since in only a quarter of the North American reference cohort an intravenous contrast agent was administered, the actual difference between BMD in the two cohorts is likely even larger. Jang et al. reported that only in patients in the age groups of <30 and 30–34 was BMD significantly higher due to intravenous contrast administration, and vice versa in the age groups of 50–54 and 60–64, yet to a smaller extent. If the former phenomenon applies to our cohort, the mean HU values of the relevant young age groups in our cohort might have been increased by contrast administration, which could explain why the mean BMD of only these young age groups was not significantly lower as compared to the values of Jang et al. Lastly, the comparison of the North American values with the Dutch cohort might be interscanner variability in HU measurements. However, Jang et al. only used multidetector CT scanners from GE Healthcare, which usually gives lower HU measurements in the same phantom as compared to Philips multidetector CT scanners used in this study [13,32]. Therefore, it is unlikely that interscanner variability impacted this study’s results.

Taking the aforementioned considerations into account, the present study strongly suggests actual differences in BMD between Dutch trauma patients and North American patients. The discrepancy between mean BMD values of the two continents was endorsed by a broad study that compared DXA-based BMD of European young adults versus American young adults, showing that the European values were also significantly lower [33].

A limitation of the current study and those by Jang et al. is that other clinical and non-clinical parameters which have been shown to affect BMD, such as weight, race, comorbidities, and smoking status, were not investigated [13,21,34,35]. Since the population size in this study is somewhat limited and only trauma patients were used, mean BMD values could have been affected by the aforementioned unknown factors. However, the ASA classification in the current study cohort suggests that considerably healthy subjects were included. Secondly, this study is limited by its retrospective study design. Therefore, the representativeness for the normal population could only be hypothesized and not tested, which may result in a selection and sampling bias. Thirdly, the proportion of female patients (32% in the study cohort) might attenuate the applicability of this cohort as a proxy for the European population. This discrepancy is most probably due to a selection bias since level-1 trauma patients were studied.

The strengths of this study were that patients were consecutively included in contrast to the North American reference cohort. Furthermore, all CTs were performed with analogous settings, and Th12 and L2 were used as substitutes for L1 with an appropriate correction in HU.

In conclusion, this study shows that CT-based bone mineral density values in a Dutch trauma population are lower than those in North America. A difference that is significant from the age group of 35 years and above. Our findings indicate that to prevent overdiagnosis of osteoporosis and osteopenia, lower CT-based bone mineral density thresholds should be used in Dutch clinical practice compared to the North American reference. Future studies should confirm these findings and establish dedicated normative values and thresholds. Other European countries are likely to benefit from similar studies that investigate normative ranges of CT-based bone mineral density in their populations.

## Figures and Tables

**Table 1:** Cohort characteristics.

Variable	Total Cohort *n* = 624
Age, median (IQR)	51 (31–68)
Range	16–95
Male, (%)	427 (68.4%)
ASA classification, (%)	
1–2	550 (88.1%)
3–4	70 (11.2%)
Missing	4 (0.6%)
BMD in HU, mean (±SD)	152 (±66)
CT vertebra, (%)	
Th12	35 (5.6%)
L1	578 (92.6%)
L2	11 (1.8%)
Intravenous contrast agent used, (%)	574 (92.0%)
Mechanism of injury, (%)	
Motor vehicle crash	201 (32.2%)
Bicycle	125 (20.0%)
High-energetic fall	95 (15.2%)
Low-energetic fall	130 (20.8%)
Other	73 (11.7%)

Abbreviations: ASA, American Society of Anesthesiologists; BMD, bone mineral density; CT, computed tomography; HU, Hounsfield Units; IQR, interquartile range; SD, standard deviation.

**Table 2:** Bone mineral density (mean HU ± SD) divided by sex in the Dutch cohort.

Age Group	Dutch Cohort		Men		Women		*p*-Value ^a^
	Group Size	Total	Group Size	Total	Group Size	Total	
**<30**	141	220 ± 43	104	214 ± 40	37	235 ± 50	**0.027**
**30–34**	33	205 ± 49	23	196 ± 52	10	224 ± 33	0.081
**35–39**	38	190 ± 48	30	181 ± 47	8	225 ± 37	**0.016**
**40–44**	30	165 ± 43	26	161 ± 44	4	192 ± 24	0.083
**45–49**	46	163 ± 41	34	157 ± 36	12	180 ± 50	0.151
**50–54**	59	154 ± 44	42	151 ± 45	17	161 ± 41	0.421
**55–59**	52	123 ± 35	37	121 ± 38	15	127 ± 29	0.579
**60–64**	45	112 ± 49	28	115 ± 57	17	107 ± 33	0.549
**65–69**	41	111 ± 40	27	111 ± 44	14	110 ± 33	0.910
**70–74**	52	96 ± 30	34	96 ± 32	18	97 ± 27	0.838
**75–79**	33	89 ± 51	20	84 ± 60	13	96 ± 34	0.473
**80–84**	28	88 ± 37	14	97 ± 42	14	79 ± 29	0.204
**>85**	26	66 ± 33	8	66 ± 35	18	66 ± 34	0.962

^a^ Student’s *t*-test; *p*-values in bold are significantly different. Abbreviations: HU, Hounsfield Units; SD, standard deviation.

**Table 3:** Bone mineral density (mean HU ± SD) by age group in the Dutch cohort and the North American reference cohort [13].

Age Group	Dutch Cohort		Reference Cohort		*p*-Value ^a^
	Group Size	Total	Group Size	Total	
**<30**	141	220 ± 43	262	226 ± 44	0.189
**30–34**	33	205 ± 49	239	210 ± 39	0.505
**35–39**	38	190 ± 48	257	206 ± 43	**0.036**
**40–44**	30	165 ± 43	165	196 ± 52	**0.003**
**45–49**	46	163 ± 41	178	188 ± 40	**<0.001**
**50–54**	59	154 ± 44	1229	175 ± 39	**<0.001**
**55–59**	52	123 ± 35	1135	159 ± 39	**<0.001**
**60–64**	45	112 ± 49	917	150 ± 39	**<0.001**
**65–69**	41	111 ± 40	1528	138 ± 54	**0.002**
**70–74**	52	96 ± 30	1143	135 ± 50	**<0.001**
**75–79**	33	89 ± 51	997	120 ± 42	**<0.001**
**80–84**	28	88 ± 37	503	113 ± 45	**0.004**
**>85**	26	66 ± 33	551	94 ± 40	**0.001**

^a^ Student’s *t*-test; *p*-values in bold are significantly different. Abbreviations: HU, Hounsfield Units; SD, standard deviation.

## Data Availability

Not applicable.

## Appendix A

**Table A1:** Linear regression analyses and correlation coefficients of bone mineral density as dependent variable in the total cohort and per sex group.

	β-Coefficient	S.E.	*t*-Value	*p*-Value	Pearson’s Correlation Coefficient (ρ; 95% CI)
**Total**					−0.77 (−0.80–−0.73)
Intercept	273.39	4.43	61.75	**<0.001**	
Age	−2.42	0.08	−29.99	**<0.001**	
**Men**					−0.73 (−0.77–−0.68)
Intercept	264.09	5.55	47.54	**<0.001**	
Age	−2.31	0.11	−21.93	**<0.001**	
**Women**					−0.85 (−0.88–−0.80)
Intercept	298.01	7.25	41.09	**<0.001**	
Age	−2.72	0.12	−22.25	**<0.001**	

*p*-values in bold are significantly different. Abbreviations: CI, confidence interval; S.E., standard error.

**Table A2:** Sex-specific differences in BMD per age group between the North American cohort and Dutch cohort.

Age Group	Women					Men				
	Dutch Cohort		Reference Cohort		*p*-Value	Dutch Cohort		Reference Cohort		*p*-Value
	Mean ± SD	n	Mean ± SD	n		Mean ± SD	n	Mean ± SD	n	
<30	235 ± 50	37	233 ± 38	143	0.790	214 ± 40	104	219 ± 48	132	0.394
30–34	224 ± 33	10	218 ± 39	131	0.637	196 ± 52	23	205 ± 41	127	0.355
35–39	225 ± 37	8	219 ± 41	151	0.686	181 ± 47	30	191 ± 38	134	0.215
40–44	192 ± 24	4	206 ± 45	161	0.537	161 ± 44	26	191 ± 51	91	**0.008**
45–49	180 ± 50	12	198 ± 38	276	0.114	157 ± 36	34	182 ± 39	151	**<0.001**
50–54	161 ± 41	17	188 ± 41	2915	**0.007**	151 ± 45	42	175 ± 36	2144	**<0.001**
55–59	127 ± 29	15	166 ± 38	2351	**<0.001**	121 ± 38	37	166 ± 39	1731	**<0.001**
60–64	107 ± 33	17	157 ± 39	1741	**<0.001**	115 ± 57	28	157 ± 38	1383	**<0.001**
65–69	110 ± 33	14	145 ± 53	1272	**0.014**	111 ± 44	27	142 ± 47	1205	**<0.001**
70–74	97 ± 27	18	139 ± 51	915	<0.001	96 ± 32	34	135 ± 43	734	**<0.001**
75–79	96 ± 34	13	120 ± 44	633	0.051	84 ± 60	20	125 ± 42	604	**<0.001**
80–84	79 ± 29	14	112 ± 46	355	**0.008**	97 ± 42	14	120 ± 41	286	**0.042**
>85	66 ± 34	18	97 ± 41	404	**0.002**	66 ± 35	8	99 ± 49	224	0.061

*p*-values in bold are significantly different. Abbreviations: BMD, bone mineral density; SD, standard deviation.
